# The ancient koji mold (*Aspergillus oryzae*) as a modern biotechnological tool

**DOI:** 10.1186/s40643-021-00408-z

**Published:** 2021-06-22

**Authors:** Ghoson M. Daba, Faten A. Mostafa, Waill A. Elkhateeb

**Affiliations:** Chemistry of Natural and Microbial Products Department, Pharmaceutical Industries Researches Division, National Research Centre, El Buhouth Street, Dokki, Giza, 12311 Egypt

**Keywords:** *Aspergillus oryzae*, Food industry, Enzymes, Secondary metabolites, Functional genomics

## Abstract

*Aspergillus oryzae* (*A. oryzae*) is a filamentous micro-fungus that is used from centuries in fermentation of different foods in many countries all over the world. This valuable fungus is also a rich source of many bioactive secondary metabolites. Moreover, *A. oryzae* has a prestigious secretory system that allows it to secrete high concentrations of proteins into its culturing medium, which support its use as biotechnological tool in veterinary, food, pharmaceutical, and industrial fields. This review aims to highlight the significance of this valuable fungus in food industry, showing its generosity in production of nutritional and bioactive metabolites that enrich food fermented by it. Also, using *A. oryzae* as a biotechnological tool in the field of enzymes production was described. Furthermore, domestication, functional genomics, and contributions of *A. oryzae* in functional production of human pharmaceutical proteins were presented. Finally, future prospects in order to get more benefits from *A. oryzae* were discussed.

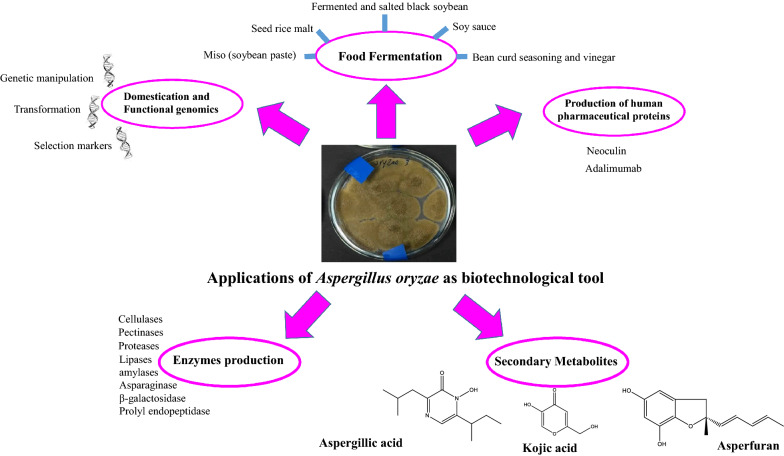

## Introduction

*Aspergillus oryzae *(*A. oryzae*) is a multicellular fungus that is considered as one of the most important species used as biotechnological tool in many countries all over the world as shown in Table 1. It is generally used in food industry for manufacturing fermented foods such as miso (soybean paste), shoyu (soy sauce), tane-koji (seed rice malt), douche (fermented and salted black soybean), bean curd seasoning and vinegar. Thanks to its potent ability to produce amylase and protease that allows it to decompose proteins and different starches into sugars and amino acids (Watarai et al. 2019). For more than 2 millennia, *A. oryzae* has been employed for koji production in the Orient. In Europe, *A. oryzae* has been used since the beginning of the previous century in enzyme production for brewing and baking (Barbesgaard et al. 1992). In the Japanese cuisine, koji is soya beans and/or cooked grain that have been fermented with a certain mold, this mold was then named koji mold to indicate the mold used for koji fermentation (Gomi 2019). *A. oryzae* has been regarded as safe (GRAS) by the FDA, and this mold safety has been approved also by the WHO (He et al. 2019). Besides its known industrial applications, *A. oryzae* has many reported biological activities that nominate it as a promising probiotic in the veterinary field for animal production (Lee et al. 2006). *A. oryzae* contributes positively in affecting gut microflora by acting as a favorable substrate for the growth of many beneficial bacteria inside intestine such as different lactobacilli species which subsequently antagonize harmful bacteria as *E. coli* and *Salmonella* due to the ability of many lactobacilli to produce bacteriocins (antimicrobial peptides) (Kim et al. 2003). Moreover, the potent enzymes produced by *A. oryzae* affect digestion process and active amylolytic and proteolytic facilitate digestion of dry matter and hence simplify getting nutrients. In a recent study, combining *A. oryzae* with date palm seed meal resulted in improving Nile tilapia’s growth, digestion activity and immunity (Dawood et al. 2020). Similarly, addition of *A. oryzae* at 0.1% to a diet resulted in a significant lowering of cholesterol levels in serum of broiler chickens for 5 weeks (Kim et al. 2003) which came as a result of the production of 3-hydroxy-3 methylglutaryl-coenzyme A responsible for inhibiting cholesterol biosynthesis (Hajjaj et al. 2005).

**Table 1:** Products of commercial values showing importance of *A. oryzae* as biotechnological tool

Product	Application	References
Dry lyophilized powder of *A. oryzae*	Probiotic; functional feed additive	Lee et al. 2006; Murphy et al. 2021
*A. oryzae* mycelia	Fermented foods industry (miso, shoyu, tane-koji, douche, bean curd seasoning, vinegar)	Taylor and Richardson 1979; Yasui et al. 2020
Amylases (α-amylases, β-amylases, and glucoamylases)	Food industry (Produced glucose during the initial stage of starch hydrolysis) Alcohol production	Chang et al. 2014 Rodriguez et al. 2006 James and Lee 1997; Biesebeke et al. 2005
Lipase	Laundry detergent	Christensen et al. 1988; Machida et al. 2008
Taka-diastase	Stomach medicine	Takamine 1894
Cellulases	Pollution treatment; animal feed, food industry; textile	Bhat et al. 2000; Chen et al. 2012
Pectinases	Juice and beverage processing, vegetable oil extraction and other food industries	Pinheiro et al. 2017
β-galactosidase (β-gal)	Food and dairy Industries	Patel et al. 1985; Furlan et al. 2000
Kojic acid	Antioxidant, whitening agent in cosmetics	Lobato et al. 2020

Contributions of *A. oryzae* have extended to the production of heterologous proteins, thanks to the prestigious secretion machinery owned by *A. oryzae* that allows it to secrete high concentrations of proteins into the culture medium (Machida, 2002). Neoculin is one of the important taste-modifying hetero-oligomeric proteins produced by *A. oryzae* (Nakajima et al. 2006). Moreover, human lysozyme, and recombinant antibodies (such as adalimumab) were also produced by *A. oryzae* in order to reduce their production costs (Huynh et al. 2020).

Due to its various industrial applications as well as its capability to produce high concentrations of proteins into the culture medium, *A. oryzae* has continuously attracted scientists’ attention as a potent biotechnological tool that has potent contributions in industrial, food, veterinary, and pharmaceutical fields. In this review, the secondary metabolites produced by *A. oryzae*, and its use as a biotechnological tool for enzymes production were presented. Moreover, domestication, functional genomics, and using *A. oryzae* for functional production of human pharmaceutical proteins were highlighted. Finally, future prospects of getting more benefits from using *A. oryzae* were discussed.

## Morphology, and classification of *A. oryzae*

**Fig. 1:**
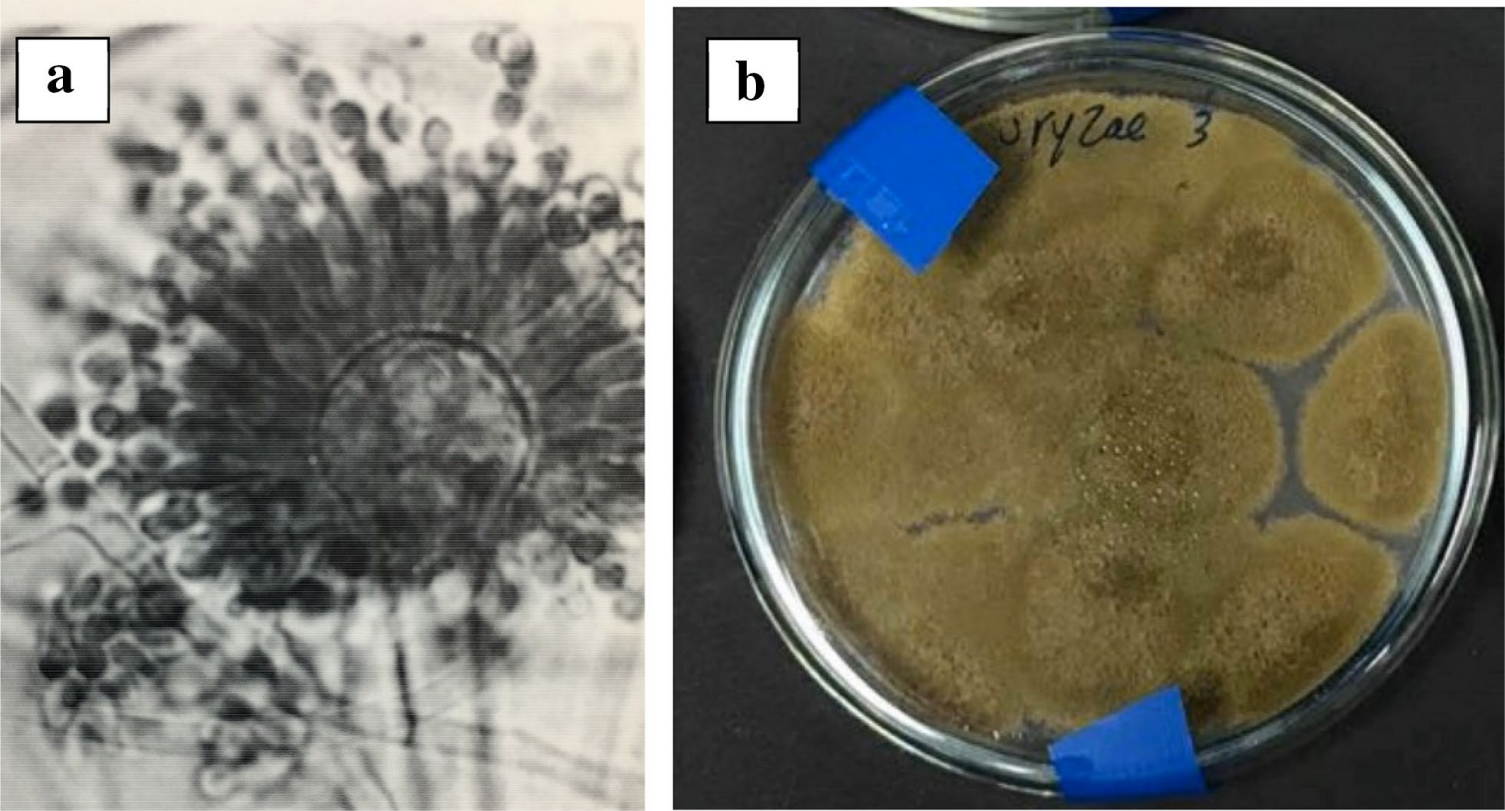
Morphology of *Aspergillus oryzae*: **A** under microscope, **B** cultivated on potato dextrose agar medium

*A.* *oryzae *belongs to *Aspergillus flavus-oryzae* group and its species can be differentiated according to their conidia shapes (Moubasher 1993; Elkhateeb 2005). *A.* *oryzae *belongs to Class: Eurotiomycetes; Order: Eurotiales; Family: Trichocomaceae. It is important to mention that molecular and genetic identification techniques usually fail to differentiate between the two fungi, *A. oryzae* and *A. flavus*, and eventually morphological and microscopic characteristics are the conclusive techniques used to identify *A. oryzae*. A. oryzae have the ability to grow on many media including, potato dextrose agar on which the growth is fast with heavy colonies, Czapek’s agar on which colonies after 7 days at 25 °C attaining 7–8 cm in diameter with faintly yellowish margin shifting to yellow green similar to the growth obtained on malt extract agar. Generally, *A. oryzae* has an optimal growth, at temperature of 32–36 °C (± 1 °C) and it cannot grow above 44 °C, in pH ranging between 5.0, and 6.0 and it can germinate at pH from 2.0 to 8.0. *A. oryzae* could grow in corn flour with a water content of about 16% (Moubasher 1993). Under microscope*, A. oryzae* is famous by its globose vesicle with elongated conidial chains, which look like fluffy-white strands on the substrate inhibited by *A. oryzae* (Moubasher 1993). *A. oryzae* conidiophores are long, arising from substrate, rough-walled, conidial head large, radiate (Fig. 1) with globose to subglobose conidia. *A. oryzae* has been isolated from soils and different plants such as rice, broad bean, sunflower, soybean, and wheat (Elkhateeb 2005).

## Secondary metabolites originated from *A. oryzae* and their bioactivities

**Fig. 2:**
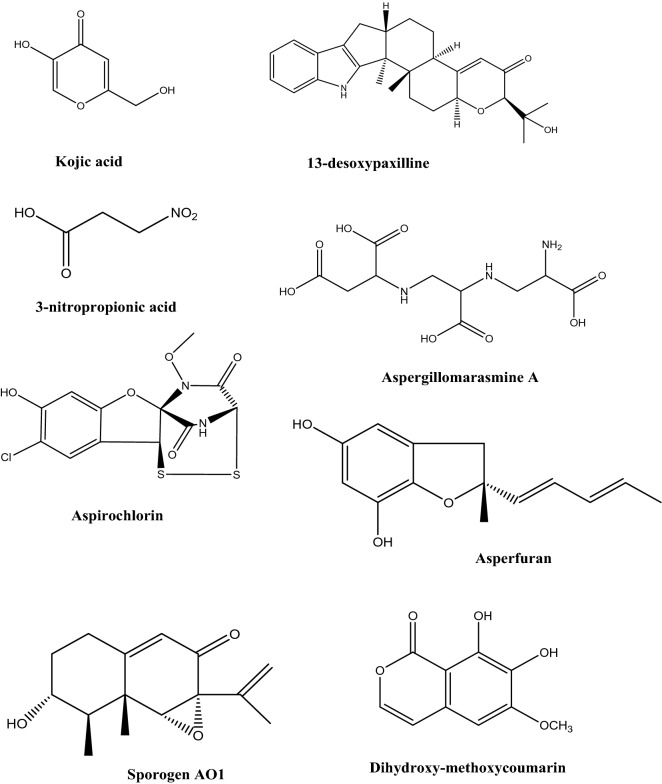

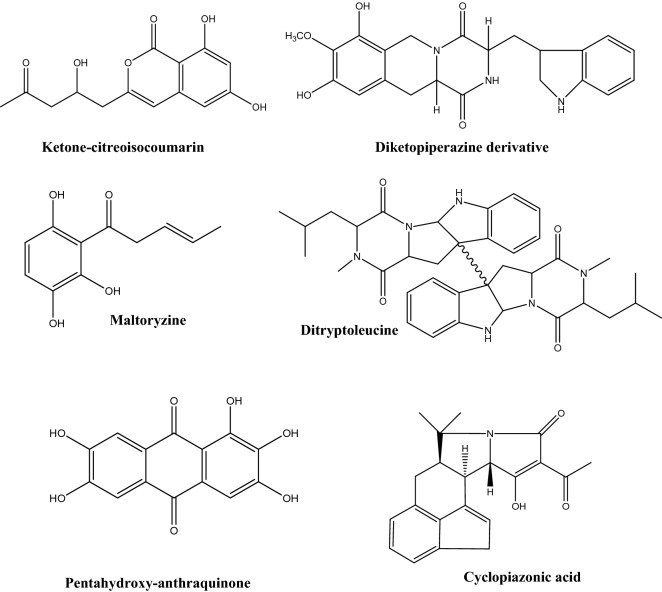

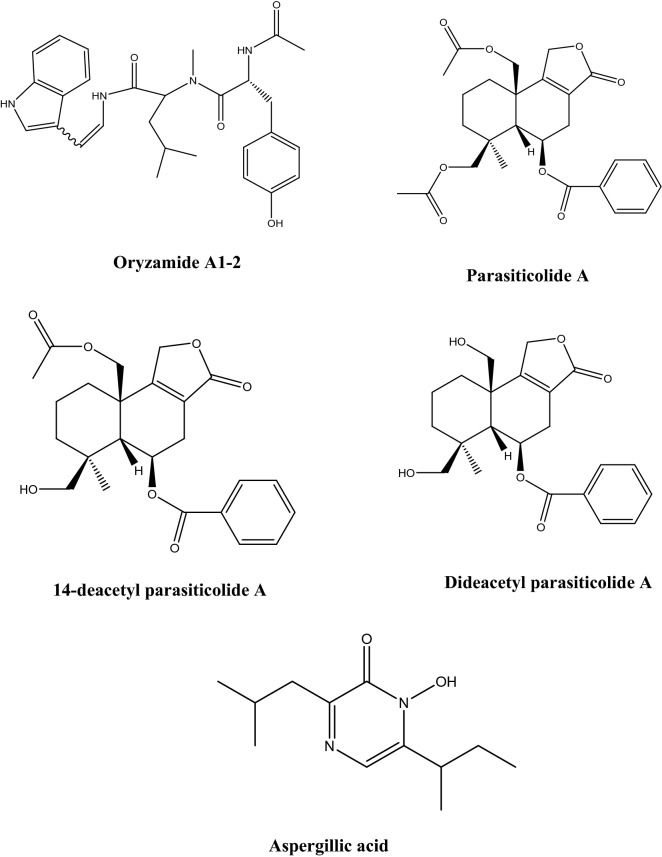
Some secondary metabolites produced by *A. oryzae*

Besides its potent secretion machinery, *A. oryzae* is a generous source of various secondary metabolites as shown in Fig. 2. Those metabolites belong to different chemical classes such as terpenoids, coumarins, oxylipins, and fatty acids (Son et al. 2018). It is important to mention that secretion of secondary metabolites in *A. oryzae* (as in case of many other microorganisms) is significantly affected by different cultivation conditions. Many of the metabolites secreted by *A. oryzae* have different reported bioactivities such as anticancer, cytotoxicity, antimicrobial, antihypertensive, and antiviral activities (Table 2). Isocoumarin derivatives produced by *A. oryzae* solid cultures exerted moderate anticancer activities against many human cancer cell lines (Zhou et al. 2016); the heterotetracyclic gliotoxin, aspirochlorine, exert antimicrobial and antifungal activities (Chankhamjon et al. 2015); aspergillic acids exhibit antihypertensive, and antifungal activities (Nishimura et al. 1991); the polyamino acid, aspergillomarasmine A is a rapid and potent inhibitor of the NDM-1 enzyme and another clinically relevant metallo-β-lactamase, and VIM-2. Aspergillomarasmine A contributed in fully restoring the effect of meropenem against many species of *Enterobacteriaceae*, *Pseudomonas*, and *Acinetobacter* (King et al. 2014). On the other hand, aspergillomarasmine B has antiangiogenic effect (He et al. 2019). Interestingly, a strain of *A. oryzae* was successfully generated to produce penicillin and the obtained strain showed over 100-fold over expression of *VeA* which is important for penicillin production (Marui et al. 2010). Asperfuran is a dihydrobenzofuran derivative that exerts antifungal and anticancer activities (Pfefferle et al. 1990). Nevertheless, some mycotoxins have also been reported from *A. oryzae*, such as kojic acid which has various applications in cosmetics as whitening agent, has strong antibacterial and anti-tyrosinase activities, and also reported to have potential carcinogenicity (Burnett et al. 2010; Saeedi et al. 2019). Also, the indol tetrameric acid, cyclopiazonic acid, and β-nitropropionic acid were also reported as metabolites from *A. oryzae* (Blumenthal, 2004). Genetic manipulation is used to remove the biosynthetic gene cluster of cyclopiazonic acid in order to avoid its biosynthesis in biotechnological processes.

**Table 2:** Some secondary metabolites produced by *A. oryzae* and their biological activities

Metabolite	Chemical class	Bioactivity and uses	References
Kojic acid	Carboxylic acid	Antimicrobial, pain killer; antioxidant; flavor enhancer, insecticide activator; has melanogenes inhibitory activity; used in skin whitening, and UV protecting products; used as iron chelator	Mohamad et al. 2010; Saeedi et al. 2019
Glutamic acid	Carboxylic acid	Protein synthesis; food additive and flavor enhancer; anticancer agent	Dutta et al. 2013
L-Malate	Carboxylic acid	Used in food and beverage industries	Ji et al. 2021
Penicillin	β-lactam antibiotics	Antimicrobial agent	Marui et al. 2010
Isocoumarins	Lactones	Anticancer activities	Zhou et al. 2016
3-Nitropropionic acid	Carboxylic acid	Neurotoxic; mitochondrial inhibitor	Geddes et al. 2000
Pyridoxine (vitamin B_6_)	Vitamin	Treat or prevent vitamin B_6_ deficiency; treat anemia; prevent or treat a certain nerve disorder	Frisvad et al. 2018
Cyanocobalamine	Vitamin	Prevent and treat vitamin B_12_ deficiency	Sakai 1953; Ramakrishnan and Sathe 1956
Aspergillic acid	Carboxylic acid	Antimicrobial and antihypertensive agent	Nishimura et al. 1991
Mutaaspergillic acid	Carboxylic acid	Growth inhibitor against hiochi-bacteria	Nakamura 1960
Aspergillomarasmine A	Polyamino acid	Metallo‐β‐lactamase Inhibitor	Zhang et al. 2017a, b
Aspirochlorin	Halogenated spiro compound	Antifungal and antibacterial activities	Rebollar-Pérez et al. 2019
Asperfuran	Dihydrobenzofuran	Antifungal and anticancer activities	Pfefferle et al. 1990
Tocopherols	Phenol	Antioxidant	Frisvad et al. 2018
Sporogen AO1	Sesquiterpene	Antifungal, antimalarial activities	Dumas et al. 2017
Phomenone	Sesquiterpene	Stimulate pro-inflammatory responses in murine cells	Rand et al. 2017

## *A. oryzae* as a biotechnological tool for industrial enzymes production

Recently, there is a major scope for utilizing low and safe resources for valuable byproducts production. Employing enzymes from microbial origin in industrial applications are preferred over enzymes produced by conventional methods due to many reasons, such as their economic feasibility, low toxicity and being eco-friendly, their low energy demand, their better efficiency, and better quality products (Gurung et al. 2013). Moreover, the competence of microbes to be cultivated on solid substrates provides the possibility of converting agricultural byproducts into valuable materials helping in both sustainable agriculture and environmental conservation. Generally, microbial enzymes of fungal origin are more favorable due to fungal hyphal mode of growth and their good tolerance to low water activity (0.5–0.6 aw), and high osmotic conditions (Raimbault 1998; Raveendran et al. 2018). Enzymes production is conducted either by solid-state fermentation (SSF) or submerged fermentation (SmF) (Subramaniyam and Vimala 2012).

Since *A. oryzae* has GRAS status (Reichelt 1983), it is safely used as a source of many industrial enzymes as shown in Table 3. Liang et al. (2009) and Chancharoonpong et al. (2012) reported high quantities of hydrolytic enzymes in soybean koji inoculated with *A. oryzae* including amylase, neutral protease, alkaline protease, metallopeptidase, and glutaminase*.*

**Table 3:** Some industrial enzymes originated from *A. oryzae* and their substrates

Enzyme	Substrate	Production yield	Reference
Neutral protease	Soy bean	84.38 U/g	Chancharoonpong et al. 2012
Alkaline protease	Soy bean	41.35 U/g	Chancharoonpong et al. 2012
	Wheat bran	600 U/ml	Ram and Kumar 2018
α-amylase, glucoamylase	Coconut oil cake	3388 U/g	Ramachandran et al. 2004
	Soy bean	200 U/g	Chancharoonpong et al. 2012
	Wheat bran	1986 U/g	Zambare 2010
	Wheat bran	36.31 U/ml	Lakshmi and Jyothi 2014
	Wheat bran and sugar cane bagasse	330 µg/ml/min	Parbat and Singhal 2011
Prolyl endopeptidases	Wheat gliadin	22 U/ml	Eugster, et al., 2015
Cellulase	Corncobs	38.80 U/ml	Sher et al. 2017
Asparaginase	Asparagine	282 U/ml	Dias et al. 2016
Lipase	Sorghum	35.66 U/ml	Ahmed et al. 2019
Pectinase	Soybean residue	120 U/ml	Meneghel et al. 2014
	Cellulose	2.03 U/ml	Ketipally and Raghu Ram 2018
β-galactosidase	Wheat bran and rice husk	386.6 U/ml	Nizamuddin et al. 2008

Proteases are a group of multifunctional enzymes that are tremendously applicable in food, pharmaceutical, medical, and biotechnological industries, accounting for about 60% of the complete enzyme market (Ramakrishna et al. 2010). Fungal proteases are characterized by their wide biochemical diversity, susceptible to genetic manipulation, high productivity, being extracellular, and hence can be easily recovered from fermentation medium (Silva et al. 2011). de Castro and Sato (2014) utilized *A. oryzae* protease produced on wheat bran by SSF for protein hydrolysis of soy protein isolate (SPI), bovine whey protein (BWP), and egg white protein (EWP) resulting in increasing natural antioxidant activity. Prolyl endopeptidase, is another enzyme that plays an important role in digestion of proline-rich proteins, can be used as an oral enzyme therapy product in patients affected by intolerance to gluten (a group of proline-rich proteins found in wheat, rye and barley) and was reported to be produced by *A. oryzae* on wheat gliadin (Eugster et al. 2015). The amylases (α-amylases, β-amylases, and glucoamylases) are one of the very useful families of enzymes in biotechnology and have the widest range of industrial applications (Rodriguez et al. 2006). Alpha amylases (E.C. 3.2.1.1., 1,4-α-D-glucan glucanohydrolase) catalyze hydrolysis of internal α-1,4 glycosidic linkage in starch, amylopectin and amylose converting them into maltose and glucose. These enzymes are critically important especially in the detergent and the food industries (Brown et al. 2013). Ramachandran et al. (2004) utilized different oil cakes which are the byproducts obtained after oil extraction, while Fadel et al. (2020) utilized wheat bran, corn flour, sugar beet pulp, sun flower for α-amylase production by *A. oryzae.* Glucoamylases are generally used in high fructose corn syrup, glucose syrup, and alcohol production (James and Lee 1997; Biesebeke et al. 2005). Lakshmi and Jyothi (2014) reported the ability of *A. oryzae* to secrete glucoamylase through growing on wheat bran*.* On the other hand, lipases, the triacylglycerol acyl hydrolases (EC 3.1.1.3) emulsify substrates and hydrolyze glycerides to free fatty acids and glycerols. Lipases are employed in the synthesis of many dairy products, oils and fats, many applications in detergents, cosmetics and medicine (Priji et al. 2016). Lipases are commonly applied in food products to enhance both aroma and flavor of cheese, yogurt, milk, and butter (Iftikhar et al. 2011). Ahmed et al. (2019) reported lipase production by *A. oryzae* on a variety of lignocellulolytic materials (wheat bran, sorghum, rice bran, wheat straw, corn cobs).

Cellulases and pectinases are responsible for hydrolyzing cellulose and pectin, respectively, into glucose. Cellulases are widely applicable in pollution treatment, animal feed, food, textile, protoplast production, genetic engineering, paper, fuel, and chemical industries (Bhat, 2000; Chen et al. 2012). Pectinase enzyme (EC. 3.2.1.15) is ranked as one of the highly important industrial enzymes that find its application in juice and beverage processing, vegetable oil extraction and other food industries (Pinheiro et al. 2017). Hoa and Hung (2013) reported the ability of *A. oryzae* to produce cellulase and pectinase on soybean residue.

β-galactosidase (EC 3.2.1.23), is also known as β-gal, that hydrolyzes lactose (milk sugar) into glucose and galactose, used for the improvement of milk and its derivatives for consumption by people with lactose intolerance, for prevention of crystallization of lactose in frozen and condensed milk products and also for the increase of the sweetening properties of lactose (Patel et al. 1985; Furlan et al. 2000). Nizamuddin et al. (2008) utilized wheat bran and rice husk as solid substrates for β-galactosidase production. Asparaginase is also another enzyme produced by *A. oryzae*, and is responsible for catalyzing asparagine hydrolysis into ammonia and aspartic acid (Olempska-Beer 2007).

## Genomics, safety, and domestication of *A. oryzae*

Generally, strains belonging to this species show variety in color and fermentation potency. However, the relationship between the capabilities of *A. oryzae* different strains and genetic factors remains not well-studied. The first report describing the whole-genome sequencing of *A. oryzae* RIB40 was published by Japanese scientists in 2005 using whole-genome shotgun approach (Machida et al. 2005). The reported 37-Mb genome was predicted to carry 8 chromosomes, comprising 12,074 genes, and encoding proteins with more than one hundred amino acid residues. Conducting comparative genomic analysis of the whole genome sequences of *Aspergillus fumigatus* and *Aspergillus nidulans* showed that *A. oryzae* genome was larger than that of both mentioned species by 7–9 Mb (about 34 and 29% larger genome sizes, respectively) (Galagan et al. 2005). Previously, it was suggested that the genes in newly acquired regions are only insignificantly expressed under normal conditions (Kobayashi et al. 2007) and most of their functions are still unidentified, especially for those genes not directly involved in fermentation. However, recently it was reported that the unique genes principally encode secretory hydrolases, stress responses, and metabolism (He et al. 2018a). One of the good databases concerned with protein as well as gene sequences of major *Aspergillus* species including *A. oryzae*, is the *Aspergillus* Genome Database (AspGD; www. aspgd.org). In this website, besides information about *A. oryzae*, analysis tools, and manually curated data derived from published scientific articles for *A. nidulans, A. fumigatus,* and *A. niger* (Arnaud et al. 2010)*.* However, this informative website needs to be updated. It should be mentioned that according to many comparative analyses, *A. flavus* and *A. oryzae* genomes showed 99.5% similarity in coding regions and hence are regarded as genetically very closely related species. Previously, *A. oryzae* has been distinguished from *A. flavus* based on morphological characteristics and toxicity (Klich 2007; Jørgensen 2007), but recently a new molecular method was suggested to distinguish between both species based on conducting genome‐wide total single-nucleotide polymorphisms (Chang 2019). Contrary to *A. oryzae*, some strains belonging to the genus *A. flavus* shows serious safety hazards due to their aflatoxin production ability (Kumar et al. 2017; Ezekiel et al. 2019). Interestingly, some *A. oryzae* strains contain all or parts of the biosynthetic gene cluster responsible for aflatoxin production, though they are non-aflatoxigenic (Kusumoto et al. 2000; Chang 2019). In a previous comparative study, *A. oryzae* was found to form a monophyletic clade derived from one clade of *A. flavus.* This was concluded based on phylogenetic analysis that was conducted on 11 genes, aflatoxin gene cluster and single-nucleotide polymorphism of the whole *A. oryzae* genome (Geiser et al. 1998; Chang et al. 2006). Although it was believed that both *A. oryzae*, and *A. flavus* have no sexual reproduction, genome analysis along with some recent studies showed that *A. flavus* can perform sexual reproduction. Moreover, both species contain an approximately complete gene set essential for sexual reproduction (Geiser et al. 1996; Wada et al. 2012). All strains of *A. oryzae* and *A. flavus* have one mating type (MAT type) locus in their genome, at which either MAT1-1 or MAT1-2 is encoded (Ramirez-Prado et al. 2008; Wada et al. 2012). Nevertheless, full sexual reproduction has not been confirmed in *A. oryzae*, and crossbreeding trials failed (Watarai et al. 2019). It was proposed by genome analysis that recombination happened among *A. oryzae* ancestors based on the disequilibrium in linkage between MAT types and a single gene phylogeny (Chang and Ehrlich 2010).

Development of the technology of third-generation sequencing has speeded the progress of genome projects concerned by *A. oryzae*, thanks to the long reads ability of this technology, which can improve genome annotation quality by reducing genome errors in assembly. Till now, the whole genome of about 10 strains of *A. oryzae* was deposited in NCBI database with genome sizes ranging between 35.42 and 41.16 Mb, and GC content of approximately 48% in most strains (He et al. 2018b). Fungal genomics is in its early stages for the breeding of industrial strains, in spite of the decoded genomes of numerous *A. oryzae* strains. Out of more than 10,000 genes, only about 200 genes (accounting for 1.7% of the whole genome) are functionally verified (He et al. 2018b, 2019).

Domestication of microorganisms in general and of *A. oryzae* in particular is attracting the attention of current researchers. Domestication is simply concerned with wild species artificial selection and breeding in order to obtain cultivated variants that fulfill human or industrial needs (Steensels et al. 2019). Domestication of microorganisms has been highly appreciated in industrial fields especially in fermentation of beverage and food. Throughout domestication process, microorganisms acquire the ability to consume particular nutrients in an efficient way, survive the specific stress during industrial process, and produce desirable compounds. Interestingly, various lineages of the same species are adapted to highly varied niches, which results in phenotypically and genetically different strains (Steensels et al. 2019). Domestication is of *A. oryzae* includes selecting strains characterized by rapid mycelial growth, pleasing fragrances, high amylases yield, and low production of toxins or pigments. In a recent study, whole-genome sequencing was conducted on 82 industrial *A. oryzae* strains to determine their draft genomes. It was suggested that *A. oryzae* strains have passed by multiple intergenomic recombination events between ancestors of *A. oryzae* without any appearance of sexual recombination throughout the process of domestication, and that domestication of *A. oryzae* is tremendously limited to intra-genomic mutation and rearrangements (Watarai et al. 2019). Moreover, conducted intra- and inter-cladal comparative study revealed that nonsynonymous, gap mutations, and intra-genomic recombination were caused by the evolutionary pressure of domestication in a selective way. The strong ability *of A. oryzae* to produce different hydrolytic enzymes nominates it as a perfect model for the research of protein secretion and gene expression (Machida et al. 2005; He et al. 2019). Nevertheless, functional genomics progress in the field of *A. oryzae* remains limited due to the small number of available selection markers for *A. oryzae*, which are not amenable to traditional genetic manipulation measures (Zhong et al. 2018). Hence, exploiting effective genetic transformation and selection markers techniques is of great importance.

## Strategies for *A. oryzae* functional genomics

**Fig. 3:**
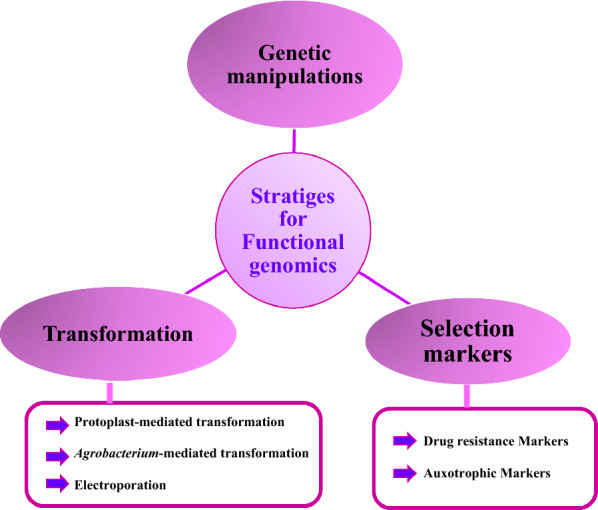
Efficient strategies for fungal transformation conducted for functional genomic investigation

Different strategies reemployed in order to investigate functional genomic as shown in Table 4 and Fig. 3, including the use of selection markers, conducting transformations, and genetic manipulations (Mei et al. 2019; He et al. 2019).

**Table 4:** Different strategies generally employed to investigate functional genomic

Strategy		Advantage	Disadvantages	Example	References
Selection markers	Drug resistance markers	Host strain can grow in presence of specific concentrations of that drug	The need for expensive antibiotics A very few number of foreign hetero gene markers can be used as markers Risk of resistance genes transfer to environment and other microorganisms	Aureobasidin resistance gene; bleomycin resistance gene; hygromycin B resistance gene	Kubodera et al. 2000; Yu et al. 2017; Zhong et al. 2018; Miura et al. 2018; Jin et al. 2021
	Auxotrophic markers	Effective, resulting auxotrophs have similar phenotype with the wild type		The gene *pyrG* encoding for orotidine-5’-monophosphate (OMP) decarboxylase	Zhu et al. 2013
Transformation	Protoplast-mediated transformation	Main strategy used to introduce DNA into fungi	Difficulty in generalizing protoplast-mediated transformation protocols across different fungi	Introducing aspartic proteinase into *A. oryzae* from *Mucor pusillus*; transforming a neutral ceramidase orthologue into *A. oryzae*	Tada et al. 2009; He et al. 2019
	*Agrobacterium*-mediated transformation	Simple and effective Fungal spores are used directly No need to obtain protoplast Can elevate gene deletion efficiency and targeted integration	Difficulty of developing enough *vir* genes and heterologous DNA containing binary vectors Difficulty in generalizing *Agrobacterium*-mediated transformation protocols		
	Electroporation	Relatively simple Relatively need short time	Not successful in case of *A. oryzae* Protocols require optimization Has a low DNA transfer efficiency		
Genetic manipulations		Successful strategy used to investigate gene functions or enhance the ability of a strain to produce a certain product	Difficulty in isolation of *A. oryzae* conidia containing only mutated nuclei Low mutational rates	CRISPR/Cas9 system	(Nødvig et al. 2015; El-Sayed et al. 2017)

### Selection markers strategy

Starting with the effective selection markers which can reduce both false-positive rates as well as screening workload, the most commonly employed selection markers are drug resistance markers and auxotrophic markers. The drug resistance markers can be used as dominant selection markers, and for those markers no need that host strain be auxotrophic. After vector transformation with drug resistance genes, the host strain is able to grow in presence of specific concentrations of that drug. However, many disadvantages are facing the use of this technique such as the need for expensive antibiotics, and the high native drug resistance nature of *A. oryzae* resulted in a very few number of foreign hetero gene markers that can successfully be used as markers (Zhong et al. 2018; Miura et al. 2018). Moreover, some resistance genes can be transferred to the environment and other microbes, which is risky. For *A. oryzae* genetic transformation, some resistance genes such as hygromycin B resistance gene (*hph*) and (*hygr*), are examples of commonly used drug resistance markers. Interestingly, by using *hph* as a selection marker, scientists have succeeded in constructing an interference vector for *A. oryzae* and repressing the *wA* gene expression which is involved in spores coloring (Fernandez et al. 2012). Also, the bleomycin resistance marker has also been employed in *A. oryzae* transformation through increasing *A. oryzae* susceptibility to bleomycin (Suzuki et al. 2009). Similarly, phleomycin and pyrithiamine resistance genes have been used for *A. oryzae* transformation as dominant selection markers (Zhang et al. 2017a, b). Anyway, due to the disadvantages of using drug resistance markers, FDA has forbidden using such markers in food-related microorganisms such as *A. oryzae* (Newsome et al. 2009). On the other hand, using auxotrophic markers as selection markers for screening is suitable and effective, based on selective culture media, transformation systems for *A. oryzae* was mainly improved according to auxotrophic markers for auxotrophic mutant strains complementation. After vector transformation with the corresponding auxotrophic marker, the resulting auxotrophs have similar phenotype with the wild type. The gene *pyrG* encoding for orotidine-5’-monophosphate (OMP) decarboxylase, which is a key enzyme for the biosynthesis of uridine/uracil that is important for fungal survival, and hence the pyrG mutants fail to convert orotidine into uridine and require additional supplementation of uridine or uracil for their growth (Zhu et al. 2013). For *A. oryzae* genetic transformation, *pyrG* is considered as the most commonly employed marker. A gene knockout system was developed using *A. oryzae* KBN630 as an original strain, and *pyrG* as a selection marker (Yoshino-Yasuda et al. 2011). Also, it was proven that the *pyrG* marker is a potent tool for recombinant gene expression investigations and genetic transformation concerned with *A. oryzae* (Nguyen et al. 2017). Moreover, multiple genes were disrupted and knocked out through a pyrG selectable marker (Maruyama and Kitamoto 2008; Yoon et al. 2011). Other nutritional markers were also investigated for *A. oryzae* transformation systems such as *adeA* for adenine, *aopex11-1* which is involved in peroxisome proliferation, *niaD* for nitrate assimilation, or *argB* for arginine biosynthesis (Jin et al. 2004a, 2004b; He et al. 2019).

### Transformations strategy

The second group of strategies employed to investigate functional genomic are those conducted for transformation of *A. oryzae*. The low *A. oryzae* transformation efficiency represents the key obstacle to its successful genetic transformation (Jiang et al. 2013). Hence, to overcome this problem, researches employed different transformation techniques including protoplast-mediated transformation (which is the main strategy to introduce DNA into fungi due to the easiness of obtaining homozygotes), *Agrobacterium*-mediated transformation, and electroporation.

#### Protoplast-mediated transformation

This strategy is the main strategy used to introduce DNA into fungi due to the easiness of obtaining homozygotes. Examples for protoplast-mediated transformation are introducing an aspartic proteinase into *A. oryzae* from *Mucor pusillus* using *niaD* gene as selective marker; transforming a neutral ceramidase orthologue into *A. oryzae* (Tada et al. 2009). Due to the variation in composition of cell wall and potential fungal defense mechanisms, it is difficult to generalize protoplast-mediated transformation protocols across different fungi (Van den Berg et al. 2015). Accordingly, the procedure used for protoplast-mediated transformation of *A. oryzae* is a modified version, where preparation of protoplast is the main step that requires cell wall removing by cell wall-lytic enzymes or other mechanical non-enzymatic methods (Wang et al. 2017). Consequently, the transformation rate in filamentous fungi is principally affected by the efficiency of cell wall-degrading enzymes (Kück and Hoff 2010).

#### The *Agrobacterium*-mediated transformation strategy

*Agrobacterium*-mediated transformation is another simple and more efficient approach for targeting gene in *A. oryzae*. Thanks to the direct use of fungal spores, and the fact that obtaining protoplasts become not necessary, which is hard and relatively difficult. Moreover, the *Agrobacterium*-mediated transformation method can elevate gene deletion efficiency and targeted integration in some fungal genera when compared with other transformation methods. The *Agrobacterium*-mediated transformation strategy was traditionally used in plants, but now it is applied to yeast and filamentous fungi (Wang et al. 2014; Nguyen et al. 2016; Li et al. 2017). The reason for using the Gram-negative bacterium, *Agrobacterium tumefaciens*, is due to its ability to transfer the T-DNA region of the Ti plasmid to the infected plant genome. This method was successfully employed for transforming fungal spores, mycelia, and germlings (Govender and Wong 2017). There are some disadvantages for using *Agrobacterium*-mediated transformation such as the difficulty of developing enough *vir* genes and heterologous DNA containing binary vectors. Also due to the difficulty in generalizing *Agrobacterium*-mediated transformation protocols across different fungi because of the variable parameters controlling *Agrobacterium*–fungi conjugation and affecting rate of transformation.

#### Electroporation strategy

The third technique of transformation is electroporation, where a high-voltage electric pulse is applied to protoplasts and DNA containing solution. Although electroporation of protoplasts has been conducted for several yeasts and fungi, researchers have failed to transform *A. oryzae* through electroporation (Timofeev et al. 2019; Lichius et al. 2020). Therefore, there are no studies reported on conducting *A. oryzae* transformation using electroporation methods. Generally, electroporation protocols require optimization among different fungal species and are relatively difficult. Additionally, although electroporation is relatively simple and need relatively short time, it has a low DNA transfer efficiency (∼1–5 × 10^4^ colonies/µg) in comparison with protoplast-mediated transformation (1 transformant per 10^5^ spores) and *Agrobacterium*-mediated transformation (10 transformants per 10^5^ spores) methods (Kotnik et al. 2015).

### Genetic manipulations

Besides selection markers, and transformation strategies discussed before, genetic manipulations is a successful strategy used to investigate gene functions or enhance the ability of a strain to produce a certain product (Son and Park 2020; Ullah et al. 2020). To do so, it is important to generate a host with effective selection markers and high transformation rate. Till now, few attempts had been performed to manipulate genes in *A. oryzae*. For instance, highly branched mutants were generated by UV or nitrous acid mutagenesis (Bocking et al. 1999). Also, a novel food-grade industrial koji molds were bred with high acid protease activity by interspecific genome recombination between *A. oryzae* and *A. niger* (Xu et al. 2011). Furthermore, an RNAi system was constructed for gene silencing in *A. oryzae* using the Gateway system and compatible restriction enzyme sites to create the hairpin RNA cassette (Yamada et al. 2007). Recently, CRISPR/Cas9 bacterial and archaeal immune mechanism was engineered into a simple, efficient, and powerful gene editing system, which contained only two components: the Cas9 nuclease and a single guide RNA (sgRNA). The CRISPR/Cas9 system has been successfully adapted to filamentous fungi such as *Aspergillus aculeatus*, *Aspergillus fumigatus*, and *A. oryzae* (Nødvig et al. 2015; El-Sayed et al. 2017). However, difficulty in isolation of *A. oryzae* conidia containing only mutated nuclei, and the low mutational rates represent obstacles against using this system for genome editing used in *A. oryzae* (Katayama et al. 2016). The procedure of CRISPR/Cas9 system starts with optimizing codon usage of cas9 then inserted into the expression vector. After that, a promoter is fused with the sgRNA sequence and inserted into the expression vector containing cas9. A CRISPR/Cas9 system in *A. oryzae* was developed to knock out *wA* (polyketide synthase), *pyrG*, and *yA* (p-diphenol oxidase) (Katayama et al. 2016).

## Functional genomics for improving *A. oryzae* industrial application

In order to improve *A. oryzae* for industrial applications, the functional genomics of *A. oryzae* can be employed mainly on conidiation, protein secretion and expression, and secondary metabolites.

### Regulation of conidiation process

**Fig. 4:**
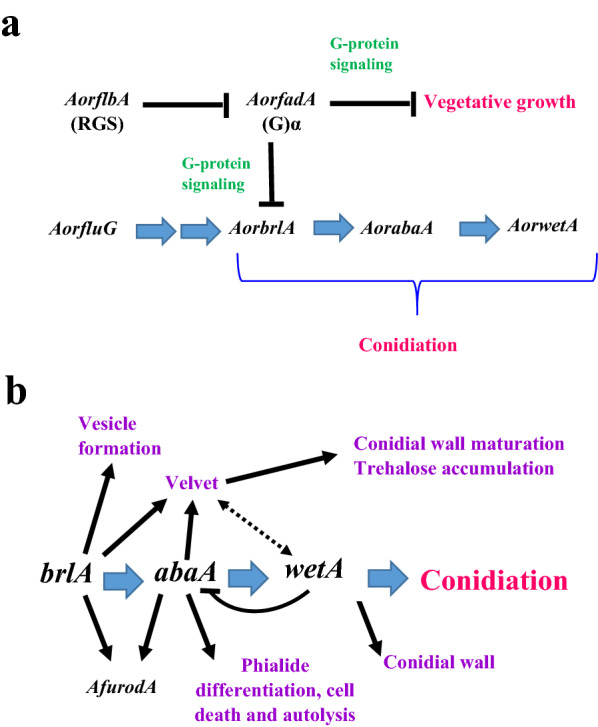
Schematic presentations showing model for conidiation regulation in *A. oryzae* as proposed by Ogawa et al., 2010 (**a**); and central regulatory pathway in *Aspergillus fumigatus* conidiation as proposed by Yu, 2010 (**b**)

Conidiation is the process of producing conidia in conidiophores (Kitamoto 2015). The conidia of *A. oryzae* are important for the food industry as starters that digest ingredients in the first step of fermentation (Ogawa et al. 2010). Hence, conidiation regulatory pathway has attracted researchers’ attention. Generally, conidiation process is putatively induced by external or internal signals that result in activation of a genetically programed sporulation (Murthy et al. 2018). Conidiation is induced when nutrients (carbon and nitrogen sources) are poorly available in culture medium (Krijgsheld et al. 2013). The regulatory mechanism of conidiation has been well-studied in *A. nidulans,* and many studies have reported that the regulatory mechanisms are conserved between *A. nidulans* and *A*. *oryzae*, and that *A. oryzae*, *A. nidulans*, and *A. fumigatus* have a G-protein signaling pathway and *brlA* orthologs in common. Moreover, the analyses of *AorflbA* disruptant and *AorfadA* dominant-active mutants implicated that *AorFadA*-mediated G-protein signaling suppresses vegetative growth of *A. oryzae* as illustrated in Fig. 4a (Ogawa et al., 2010). Till now, *BrlA* is the most well-studied conidiation regulatory gene. *BrlA* triggers a central regulatory pathway (Fig. 4b) regulating conidiation-specific genes expression such as *abaA*, *medA, stuA*, *vosA,* and *wetA* (Park et al. 2019). *BrlA* disruptants of *A. oryzae* failed to form conidiophores. On the other hand, the fadA G-protein-dependent signaling pathway also regulates conidiation by suppressing *brlA* activation. The Fad-mediated signaling is regulated by FlbA, which is a specific regulator of G-protein signaling. In *A. oryzae,* the fadA mutant is responsible for reducing intrinsic GTPase activity and causes formation of autolytic phenotypes. Furthermore, the overexpression of Rim15p (a serine-threonine kinase) in *A. oryzae* caused reduction in conidiation process which is completely stopped when Rim15p is deleted (Nakamura et al. 2016).

### Protein and secondary metabolites secretion and expression

Being listed as GRAS by the FDA, and due to its potent secretion machinery, *A. oryzae* is an excellent host for industrial protein production such as amylases, chymosin, glucose oxidases, cellulases, lipases, pectinases, catalases, proteases, phytases, and xylanases in food (He et al. 2018a; Ntana et al. 2020). As a result, *A. oryzae* has been attracting continuous attention as a host for expressing homologous and heterologous proteins. Nevertheless, heterologous eukaryotic proteins are usually inefficiently produced compared to endogenous proteins. Till now, numerous obstacles have been reported and solved in the heterologous protein production process. Firstly, the most important factors are effective selection markers and effective *A. oryzae* transformation strategies, which have been described previously. Secondly, presence of secreted proteases with their proteolytic degradable abilities in the culture medium is considered as another serious obstacle in heterologous protein production (Yokota et al. 2017). To overcome this proteolytic degradation, scientists have constructed multiple protease gene disruptants, which increased the heterologous proteins yields (Yoon et al. 2011; Hoang et al. 2015). Moreover, a significant improvement of heterologous protein production by *A. oryzae* is accomplished by repressing autophagy genes and vacuolar protein sorting (VPS), which play key roles in the secretory pathway (Yoon et al. 2010). Furthermore, the yield of heterologous proteins was improved through genetic fusion of a target protein with endogenous carrier proteins which are usually secreted enzymes (Yoon et al. 2010). In transcription and posttranscriptional processes, heterologous fusion protein technique can improve yields. The approach of fusion proteins was recently developed to increase heterologous protein expression through the modulation of endoplasmic reticulum–Golgi cargo receptors (Hoang et al. 2015). The carrier-fused heterologous proteins secretion can be affected by lectin-type cargo receptors and hence improve heterologous protein production.

Comparing the *A. oryzae* genome with those of *A. fumigatus* and *A. nidulans* revealed that extra genes are predominantly involved into secretory hydrolases, transporters, and secondary metabolites production genes. Generally, secondary metabolites are synthesized by gene clusters, most of which encode typical backbone enzymes, such as non-ribosomal peptide synthase and polyketide synthase. The *A. oryzae* genome contains 56 gene clusters, from which only 14 genes are identified as biosynthetic gene cluster involved in production of kojic acid. These genes include an enzyme gene (*kojA*), Zn(II)2Cy6 transcription factor gene (*kojR*), and a transporter gene (*kojT*) (Yamada et al. 2014). The safety of *A. oryzae* can be explained by the lack of the *aflR* gene which made *A. oryzae* cannot produce aflatoxins (Lee et al. 2014).

## Functional production of human pharmaceutical proteins using *A. oryzae*

*A. oryzae* has been nominated as a promising host for production of heterologous proteins from different high eukaryotes due to its ability to secrete high concentrations of proteins into the culture medium (Machida 2002). Furthermore, using *A. oryzae* for the production of the hetero-oligomeric protein, neoculin, which has taste-modifying activity was also reported (Nakajima et al. 2006).Tsuchiya et al. in (1992) have reported production of active human lysozyme from *A. oryzae* transformants through expression of the introduced mature human lysozyme (HLY gene). Moreover, the great demand together with the requirement for reducing cost of antibodies has attracted serious attention in order to find a suitable expression platform for producing recombinant antibodies. In a recent study, *A. oryzae* was employed for the production of adalimumab, which is an antibody (IgG) that binds in a specific way to the inflammatory cytokine, human TNFα. Adalimumab has been used in the treatment of some chronic inflammatory diseases such as rheumatoid arthritis. Production of adalimumab was successfully conducted in the culture supernatant of *A. oryzae* transformants with comparable affinities and bioactivities to its commercial counterpart (Huynh et al. 2020).

## Conclusion and future prospects

Due to its various applications in food, veterinary, and pharmaceuticals industries, *A. oryzae* is considered as a potent biotechnological tool of great interest. Recent advances in techniques such as next-generation sequencing has improved the research on the functional genomics of this valuable fungus, which is helpful to the genetic enhancement of *A. oryzae* fermentative strains. Coupling of protoplast-mediated transformation along with *Agrobacterium*-mediated transformation will contribute in enhancing transformation process. Discovering new strategies, and optimization of current strategies used for functional genomics of *A. oryzae* will be helpful for getting maximum benefits from *A. oryzae* in order to be employed in industrial production. Finally, updating and developing current websites, and creating new open access websites carrying recent literature about *A. oryzae* is of great importance. Such tools can facilitate researchers work and keep them updated with recent available data concerning this industrially important koji mold.

## Data Availability

Not applicable.
